# Thyroid Autoantibodies Display both “Original Antigenic Sin” and Epitope Spreading

**DOI:** 10.3389/fimmu.2017.01845

**Published:** 2017-12-20

**Authors:** Sandra M. McLachlan, Basil Rapoport

**Affiliations:** ^1^Thyroid Autoimmune Disease Unit, Cedars-Sinai Medical Center, UCLA School of Medicine, Los Angeles, CA, United States

**Keywords:** thyroid autoantibodies, intermolecular and intramolecular epitope spreading, immunodominant region, original antigenic sin, thyroglobulin, thyroid peroxidase, thyrotropin receptor

## Abstract

Evidence for original antigenic sin in spontaneous thyroid autoimmunity is revealed by autoantibody interactions with immunodominant regions on thyroid autoantigens, thyroglobulin (Tg), thyroid peroxidase (TPO), and the thyrotropin receptor (TSHR) A-subunit. In contrast, antibodies induced by immunization of rabbits or mice recognize diverse epitopes. Recognition of immunodominant regions persists despite fluctuations in autoantibody levels following treatment or over time. The enhancement of spontaneously arising pathogenic TSHR antibodies in transgenic human thyrotropin receptor/NOD.*H2^h4^* mice by injecting a non-pathogenic form of TSHR A-subunit protein also provides evidence for original antigenic sin. From other studies, antigen presentation by B cells, not dendritic cells, is likely responsible for original antigenic sin. Recognition of restricted epitopes on the large glycosylated thyroid autoantigens (60-kDa A-subunit, 100-kDa TPO, and 600-kDa Tg) facilitates exploring the amino acid locations in the immunodominant regions. Epitope spreading has also been revealed by autoantibodies in thyroid autoimmunity. In humans, and in mice that spontaneously develop autoimmunity to all three thyroid autoantigens, autoantibodies develop first to Tg and later to TPO and the TSHR A-subunit. The pattern of intermolecular epitope spreading is related in part to the thyroidal content of Tg, TPO and TSHR A-subunit and to the molecular sizes of these proteins. Importantly, the epitope spreading pattern provides a rationale for future antigen-specific manipulation to block the development of all thyroid autoantibodies by inducing tolerance to Tg, first in the autoantigen cascade. Because of its abundance, Tg may be the autoantigen of choice to explore antigen-specific treatment, preventing the development of pathogenic TSHR antibodies.

## Introduction

The concept of “original antigenic sin” arose from findings in humans and mice infected with influenza virus (1, 2) and in mice responding to Chlamydia proteins (3). For example, in sequential immunization of mice with two antigenically related but different strains of influenza A virus, antibodies induced by the second infection reacted more strongly with the primary than with the secondary virus (1). Similarly, humans infected with a novel influenza virus expanded antibodies against a viral strain of a previous infection and failed to develop antibody responses to epitopes on the new viral strain (4). Unlike in viral infections, the presently held authoritative opinion is that autoimmunity involves the converse of the “doctrine” of original antigenic sin, thereby facilitating “an unforseen platform for immune therapy” (5). In this review, we present contrary evidence supporting the concept of an original antigenic sin component occurring for autoantibodies in thyroid autoimmunity and perhaps for other autoantibody-mediated diseases.

A phenomenon interlinked with original antigenic sin is epitope spreading, a well-recognized feature of some autoimmune conditions such as type 1 diabetes mellitus and multiple sclerosis, as well as for the animal models of these diseases, namely NOD mice and experimental autoimmune encephalomyelitis (EAE). Spreading can involve increasing the number of epitopes recognized on the same autoantigen (intramolecular) and subsequent recognition of additional autoantigens (intermolecular) over time. An example of intermolecular spreading, in EAE, SWX mice immunized with myelin proteolipid protein (PLP) develop the (as expected) T cell reactivity to determinants on PLP and to determinants on myelin basic protein and myelin oligodendrocyte glycoprotein (6). Similarly, in type 1 diabetes that develops spontaneously in NOD mice, T cell recognition of islet autoantigens spreads from proinsulin to other islet autoantigens such as islet-specific glucose-6-phosphatase catalytic subunit-related protein (IGRP) [for example, Ref. (7, 8)].

Thyroid autoimmune disease, Hashimoto’s thyroiditis, and Graves’ disease are the most common organ-specific autoimmune diseases affecting humans, far more common than type 1 diabetes mellitus and multiple sclerosis. In particular, approximately 1% of the population will develop Graves’ disease in their lifetime and ~15% of adult females have autoimmune thyroiditis, although usually subclinical (9, 10). Animal models (induced and spontaneous) are available that provide insight into thyroid autoimmunity in humans. Three thyroid-specific autoantigens are targeted by the immune system: abundant soluble thyroglobulin (Tg), the much less abundant membrane-bound protein thyroid peroxidase (TPO), and the thyrotropin receptor (TSHR) [reviewed in Ref. (11)]. Although the great majority of Hashimoto patients have autoantibodies to TPO and many have Tg autoantibodies, hypothyroid patients with seronegative Hashimoto’s disease have been reported (12).

Whether autoantibodies to TPO and Tg play a role in thyroid cell destruction is unclear, but they are excellent markers of the immune response to the thyroid. Moreover, B cells are increasingly recognized as powerful antigen-presenting cells by means of their membrane-bound antibodies that capture small amount of antigen for processing and presentation to T cells [for example, Ref. (13)]. TPO autoantibody-mediated and -modulated presentation to T cells has been reported (14, 15). Also, the enhancing or suppressing effects of Tg antibodies on the processing of a pathogenic T cell epitope on Tg have been described (16). Importantly, the successful treatment of Graves’ ophthalmopathy patients with a monoclonal antibody to B cells (ritixumab) was suggested to involve antibody presentation by B cells (17). Turning to responses to the TSHR, stimulating TSHR autoantibodies are the direct cause of hyperthyroidism in Graves’ disease [reviewed in Ref. (18)] and blocking TSHR autoantibodies are responsible for hypothyroidism in rare patients [for example, Ref. (19)].

Here, we examine evidence involving thyroid autoantibodies for both *intramolecular* original antigenic sin and *intermolecular* epitope spreading in autoimmune thyroid disease. Our findings have important implications for understanding disease pathogenesis and for developing novel antigen-specific therapeutic approaches to control the development of thyroid autoantibodies and thereby prevent thyroid autoimmunity rather than treating the clinical disease.

## Autoantibody Recognition in Humans of an Immunodominant Region (IDR)—A Reflection of Original Antigenic Sin

### Antibody Recognition of an IDR on Tg and TPO

**Figure 1:**
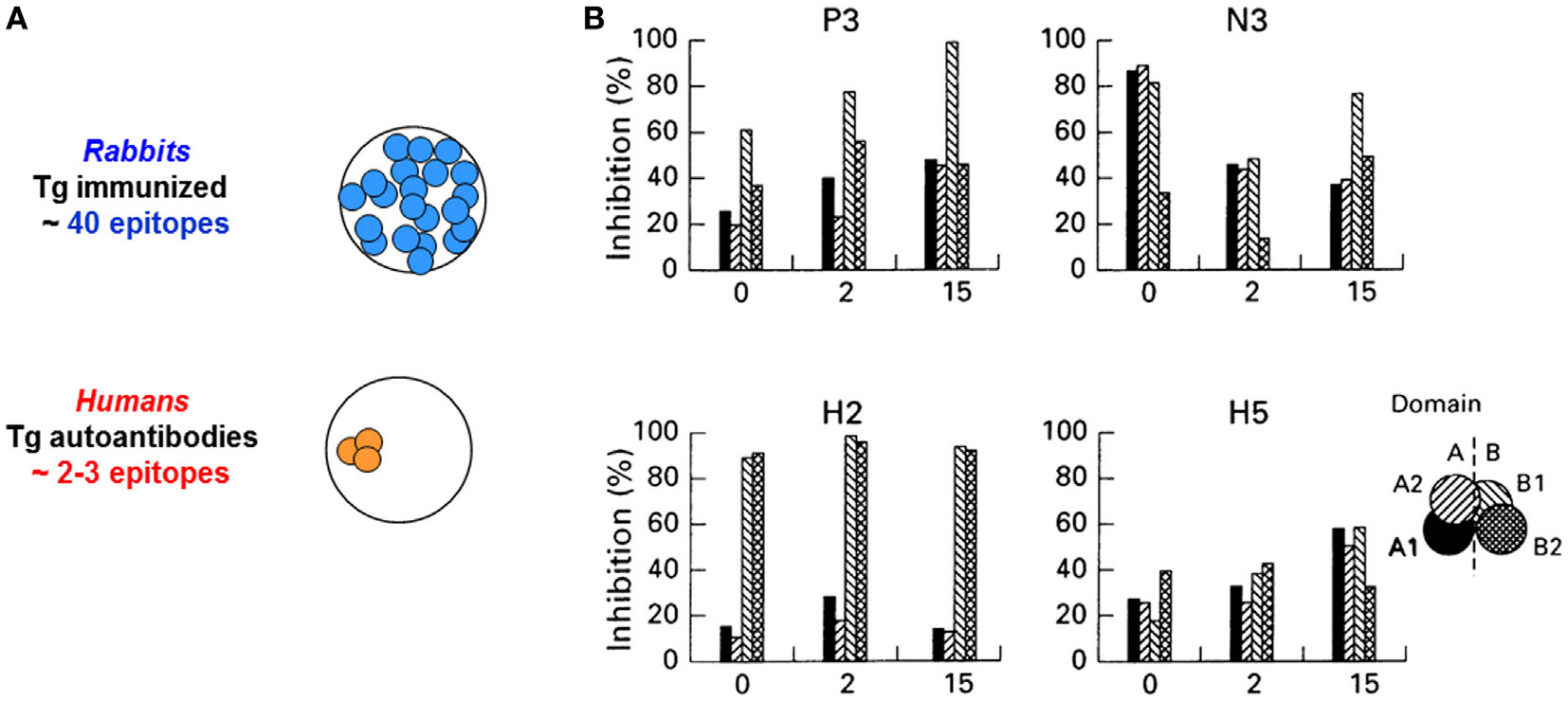
**(A)** Diverse antibody epitopic recognition in rabbits immunized with human thyroglobulin (Tg) versus restricted antibody recognition by human Tg autoantibodies in humans. Schematic illustration of the concept described in Ref. (20–23). **(B)** Recognition of thyroid peroxidase autoantibody epitopes (“fingerprints”) is stable over 15 years. The inset provides the key to the A and B domains (and subdomains) recognized by autoantibodies in the sera. Adapted from Ref. (30).

It has long been recognized that antibodies induced experimentally to Tg interact with multiple, widely diverse epitopes on the large (600 kDa) dimeric Tg molecule, whereas human Tg autoantibodies interact with a restricted number of epitopes (Figure 1) (20–23). Similar observations have been made for TPO (24, 25). Panels of human monoclonal TPO and Tg autoantibodies isolated from combinatorial immunoglobulin libraries and expressed as recombinant Fab confirmed restricted epitope recognition [for example (26, 27)]. Recombinant Fab provided the tools to characterize the IDRs on TPO and Tg recognized by antibodies in patients and some euthyroid controls with subclinical disease (Table 1). Characterization of the epitopes recognized by TPO and Tg autoantibodies has also been performed by competition assays using mouse monoclonal antibodies generated (for example) to Tg polypeptides (28), to purified TPO (25), or to TPO peptides (25, 29).

**Table 1:** Recognition of an autoantibody immunodominant region (IDR) on thyroglobulin and thyroid peroxidase (TPO) in spontaneous thyroid autoimmunity (humans and a mouse model) versus thyroid antibodies induced in rabbits or mice that are either restricted or not restricted to an IDR.

	Thyroid autoAb	Recognition	Reference
**Humans (spontaneous)**			
AITD	TPOAb	IDR	(31–33)
Normal, elderly women	TPOAb	IDR	(34)
Postpartum thyroiditis	TPOAb	IDR, stable over time	(35)
Juvenile HT; Amish HT	TPOAb	IDR, stable over time	(30, 36)
HT twins	TPOAb	IDR, stable over time	(37)
Thyroiditis; also after I_2_	TgAb	IDR	(27, 38–41)
Differentiated thyroid cancer	TgAb	IDR	(42)
Subacute thyroiditis	TgAb	IDR-B	(43)
GD treated with ^131^Iodine	TgAb	IDR	(44)
**Mice (spontaneous)**			
Thyrotropin receptor (hTSHR)/NOD.*H2^h4^* injected with TSHR A-subunit	TSHR-Ab: pathogenic	Expanded by “inactive” antigen	(45)
**Mice rabbits (induced)**			
AKR/J-mice TPO fibroblasts	TPOAb	IDR	(46)
AKR/J-mice TPO + complete Freund’s adjuvant (CFA)	TPOAb	Not restricted	(46)
Rabbits Tg + CFA	TgAb	Not restricted	(20)

It should be emphasized that binding by the majority of patients’ autoantibodies to TPO is decreased following protein denaturation (47, 48), demonstrating that most TPO autoantibodies interact with epitopes on conformationally intact protein. However, some studies have shown interaction between serum autoantibodies and reduced and denatured TPO [for example, Ref. (49, 50)], suggesting that not all TPO epitopes are conformational. Further, several linear epitopes (51, 52) and polypeptide fragments (53–56) are recognized by some patients’ TPO autoantibodies. Similarly, Tg autoantibodies predominantly recognize native protein [for example, Ref. (57)]. However, recognition of peptide fragments by some Tg autoantibodies has been reported [for example, Ref. (38, 58)].

In this review, because of their dominance in the patient repertoire, we will focus on autoantibodies to TPO and Tg that interact with conformational epitopes.

### Autoantibody Recognition of TPO and Tg Is Stable over Time

TPOAb epitopic “fingerprints” of the IDR are stable over time, that is *without* intramolecular epitope spreading, for example, in families in which the proband has juvenile Hashimoto’s thyroiditis (30), women who develop postpartum thyroiditis (35), and for TgAb in most patients on iodine supplementation (39) (Table 1). Similarly, after iodine-131 treatment in Graves’ disease, there was no evidence of TgAb intramolecular antibody spreading (44). There is some evidence that TPOAb epitopic patterns are inherited in families (36, 37).

### TSHR Autoantibody Recognition

Changes are rare in the epitopes recognized by TSHR antibodies, namely thyroid-stimulating antibodies (TSAbs), which are responsible for hyperthyroidism in Graves’ disease, and TSH-blocking antibodies (TBAbs), which cause hypothyroidism. In rare patients, TSHRAb switching from TSAb to TBAb (or vice versa) has been observed [reviewed in Ref. (59)]. The derivation of monoclonal TSAb and TBAb from one blood sample of an unusual patient who alternated between hyperthyroidism and hypothyroidism (60) demonstrated that the contrasting serum biological activities were due to two distinct antibodies.

### IDR Recognition by TPOAb Induced in Mice

It is of interest that injecting mice with TPO expressed together with MHC class II on a fibroblast line induced TPOAb that resembled autoantibodies from Hashimoto or Graves’ patients in terms of a high affinity for TPO and recognition of an IDR (46). In the same mouse strain, conventional immunization with TPO protein and adjuvant induced antibodies with lower affinity that recognized diverse epitopes on TPO (Table 1).

### Other Autoantibody Recognition of an IDR

Autoantibodies interact with epitopes in an IDR in other autoantibody-mediated diseases [reviewed in Ref. (61)]. For example, in myasthenia gravis, autoantibodies to the acetylcholine receptor are restricted to a major IDR on the α1 subunit (62). Similarly, in the skin blistering diseases such as pemphigus vulgaris and pemphigus foliaceous, autoantibodies interact with an IDR on desmoglein (63).

Overall, the stability of human thyroid autoantibody recognition of an IDR is suggestive of “original antigenic sin.” It is possible that this concept may also apply to other human autoantibodies directed to an IDR on their respective autoantigens.

## Evidence for Original Antigenic Sin in a Mouse Model of Thyroid Autoimmunity

In mice *induced* to develop Graves’ disease by immunization with an adenovirus encoding the TSHR A-subunit gene, pretreatment with a non-pathogenic (or “inactive”) form of TSHR A-subunit protein attenuated hyperthyroidism by diverting pathogenic TSHR antibodies to a non-functional variety (64). Subsequently, pathogenic TSHR antibody diversion was attempted using the same approach in a mouse model that *spontaneously* develops pathogenic TSHR autoantibodies, human thyrotropin receptor (hTSHR)/NOD.*H2^h4^* mice with the human TSHR A-subunit transgene targeted to the thyroid (65). Unexpectedly, in an example of original antigenic sin, rather than attenuating the pre-existing pathogenic TSHRAb level, injecting “inactive” TSHR A-subunit protein into hTSHR/NOD.*H2^h4^* mice enhanced the levels of pathogenic TSH-binding inhibition and TSAbs, as well as increasing the levels of non-pathogenic antibodies detected by ELISA (45). This effect was TSHR specific as spontaneously occurring autoantibodies to Tg and TPO were unaffected.

**Figure 2:**
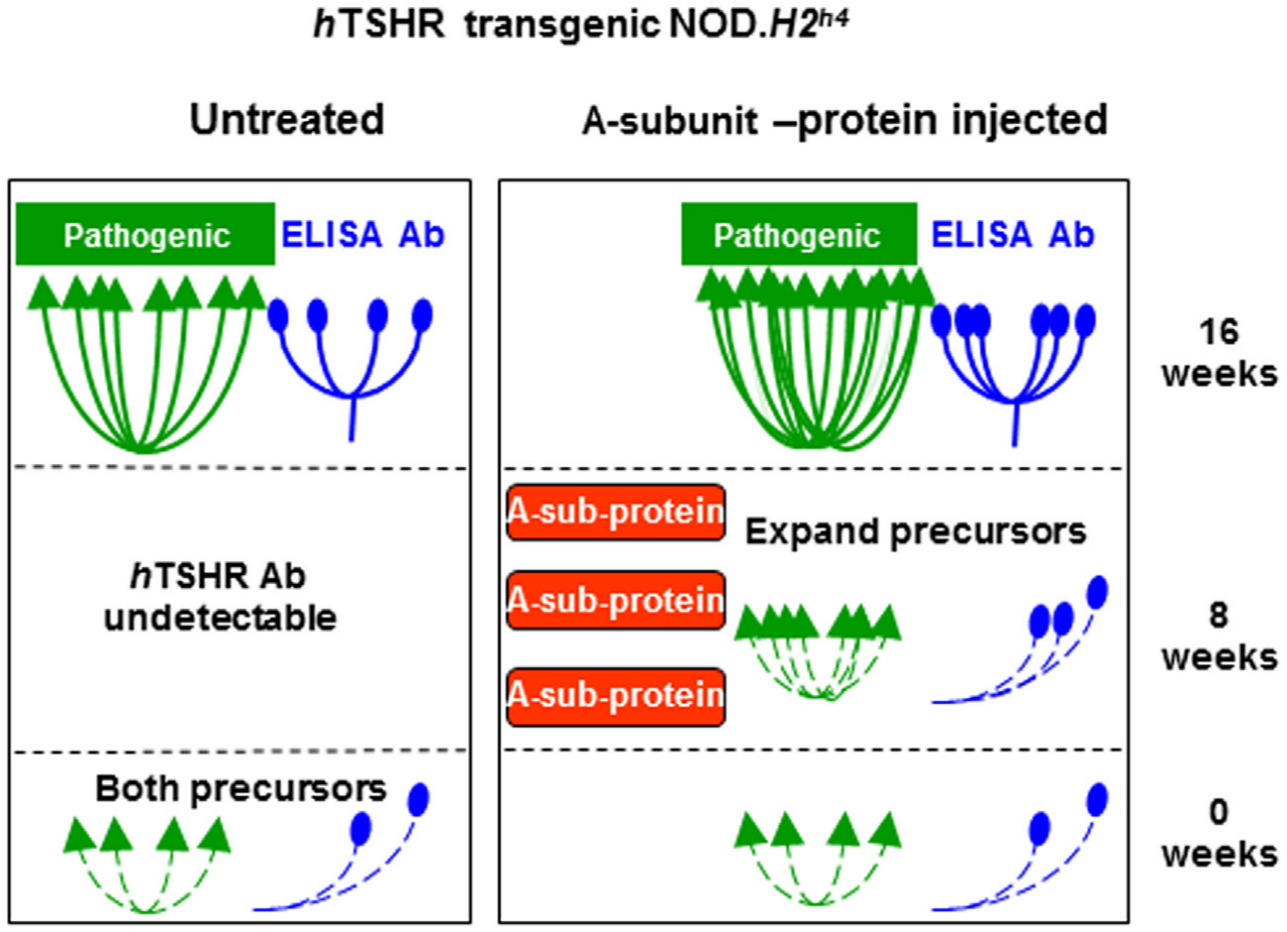
“Original antigenic sin” observed in human thyrotropin receptor (hTSHR)/NOD.*H2^h4^* mice injected with an inappropriate form of thyrotropin receptor A-subunit or saline (as control). See text for explanation. Adapted with permission from Ref. (45).

In hTSHR/NOD.*H2^h4^* mice, the original antigenic sin is the initial selection of B cells for the transgenically expressed TSHR protein, namely precursors specific for both non-functional antibodies (detectable only by ELISA) as well as pathogenic TSHR antibodies (detectable only in functional assays) (Figure 2). B cells with affinity for self antigens (like the transgenic hTSHR) are tolerized by a number of mechanisms including receptor editing and anergy (functional unresponsiveness) rather than deletion as for self-reactive T cells (66). By using transgenic hen egg lysozyme-specific transgenic mouse models, it was demonstrated that self-reactive B cells were not eliminated when this antigen was expressed by thyroid cells (67). Similarly, in the spontaneous hTSHR/NOD.*H2^h4^* model, two types of precursor B cells for pathogenic and non-pathogenic TSHRAb remain in the repertoire and both can be expanded by the “cross-reacting” antigen, the non-pathogenic TSHR A-subunit protein.

## Mechanisms and Implications of Original Antigenic Sin in Thyroid Autoimmunity

### Mechanisms of Original Antigenic Sin

The mechanisms responsible for original antigenic sin are not fully understood. However, because of the problems caused for vaccination against novel viral strains, approaches have been used to overcome original antigenic sin. These studies provide insight into the basis for this phenomenon, at least from the perspective of T cell epitopes.

One approach involves eliciting cross-reactive responses by immunization with multiple peptide variants (68) or injecting yeasts carrying diverse virus-like particles (69). It has also been suggested that, at least for CD8+ T cell responses, original antigen sin can be overcome by treatment with neutralizing interleukin 10 together with linked T cell epitopes (70). A different strategy involves immunization with adjuvants (such as *Bordetella pertussis* toxin) that activate dendritic cells (71). The latter approach is of interest in the context of comments by Kim et al. (4) concerning viral antibodies, namely that antigen presentation by B cells favors the activation of memory B cells specific for the first virus rather than naive B cells specific for the subsequent cross-reacting virus. Finally, a simple mechanistic explanation proposes that original antigenic sin occurs because regulatory T cells induced by the first antigen decrease the amount of the second antigen on dendritic cells that activate naive B cells (72).

### Implications of Original Antigenic Sin in Thyroid Autoimmunity

The most likely explanation for original antigenic sin in thyroid autoantibodies appears to involve antigen presentation by B cells or (in the case of TPO) thyroid cells, rather than dendritic cells. It should be noted that a restricted antibody response focused on the TPO IDR (as in humans) occurs in AKR/J-mice injected with cells expressing human TPO but not in mice of the same strain immunized with human TPO protein and adjuvant (46). The restricted response is likely related to the much lower concentration of cell-associated TPO than TPO injected with adjuvant. Because of their efficiency as specific antigen-presenting cells [for example, Ref. (13)], B cells are likely the antigen-presenting cells in spontaneously arising autoantibodies to Tg and the TSHR A-subunit. *Via* their specific immunoglobulin receptors, B cells may also capture conformationally intact thyroid autoantigens, a critical factor for the induction of pathogenic TSHR antibodies [reviewed in Ref. (73)].

In addition to suggesting the likely cells involved in autoantigen presentation, IDR recognition simplifies what would otherwise be a gargantuan task, namely exploring the regions recognized by autoantibodies on large, glycosylated thyroid autoantigens:

(i) Tg, the largest thyroid autoantigen comprising a homodimer of 300 kDa molecules, poses a major challenge. Iodination of Tg alters recognition by human autoantibodies (74), possibly by denaturing the antigen. However, epitope mapping using Tg fragments (fusion proteins or digestion products) suggests that some Tg autoantibody epitopes are located in the central region (75) or at the *C*-terminal end of the molecule (76, 77).
(ii) TPO is a homodimer (each 110 kDa) inserted in the plasma membrane, and TPO autoantibodies are directed against the ectodomain. The TPO epitope of an human autoantibody (expressed as a recombinant Fab) was identified using footprinting technology (78). In addition, progress has been made in delineating the amino acids targeted by other autoantibodies [for example, Ref. (79–81)]. However, definitive mapping of autoantibodies epitopes will require crystallization of a TPO monoclonal autoantibody with TPO protein.
(iii) The TSHR, like TPO, is also membrane bound but pathogenic TSHR autoantibodies (as in Graves’ disease) are induced spontaneously to the heavily glycosylated TSHR A-subunit (~60 kDa) shed after cleavage of the membrane-bound receptor [reviewed in Ref. (73)]. Amino acid residues involved in the binding sites of TSAb were initially explored using chimeric TSHR-luteinizing hormone receptors together with mutagenesis [for example, Ref. (82)]. More recently, the epitopes for monoclonal human (M22) and a monoclonal human TBAb (K1-70) have been determined by co-crystalizing each antibody with the major portion of the TSHR A-subunit (83, 84).

## Epitope Spreading of Thyroid Autoantibodies

### Intermolecular Spreading

Induced and spontaneous intermolecular spreading for thyroid autoantibodies has been demonstrated for thyroid autoimmunity (Table 2). For example, rabbits immunized with human Tg and complete Freund’s adjuvant develop antibodies to Tg, as expected, as well as antibodies to TPO and antibodies that bind to both Tg and TPO (85, 86). It should be noted that searching for previously postulated bispecific human autoantibodies that recognize both Tg and TPO (“TgPO antibodies”) [for example, Ref. (87)] in a phage display immunoglobulin gene combinatorial, constructed from thyroid-infiltrating B cells of a patient with library serum TgPO-like autoantibody activity, led to multiple antibodies specific for *either* Tg or TPO but none had TgPOAb activity (88).

**Table 2:** Thyroid antibodies: induced or spontaneous intermolecular or intramolecular antigenic spreading.

Strains/disease	Treatment	First Ab	Second Ab	Reference
**Induced—intermolecular spreading**				
Rabbit	Tg peptide + CFA	Peptide Ab	hTgAb, mTgAb	(85)
Rabbit	Tg/Tg peptide + CFA	TgAb	TPOAb, TgPOAb	(86)
HLA-DR3	hTSHR-DNA	hTSHR Ab	mTg	(89)
BALB/c hTSHR A-	Anti-CD25, hTSHR	hTSHR Ab	mTg, mTPO	(90)
Subunit (Lo-expressor)	A-subunit-adenovirus			
**Spontaneous—intermolecular spreading**				
NOD.*H2^h4^* mice	No Tx; time	TgAb	TPOAb	(91)
Juvenile HT	No Tx; time	TgAb	TPOAb	(91)
HT	No Tx; time	TgAb	TPOAb	(92)
GD	No Tx; time	TgAb, TPOAb	TSHRAb	(92)
Hyper to hypo		TSAb	TBAb	(93, 94)
Hypo to hyper	L-T4	TBAb	TSAb	(95, 96)
**Spontaneous—intramolecular spreading**				
HT	Iodine prophylaxis	TgAb	TgAb-B epitope	(97)

*CFA, complete Freund’s adjuvant; h, human; m, mouse; HT, Hashimoto thyroiditis; hTSHR, human thyrotropin receptor; GD, Graves’ disease; TBAb, TSH-blocking antibody; Tg, thyroglobulin; TSAb, thyroid-stimulating antibody*.

Returning to intermolecular epitope spreading, transgenic HLA-DR3 mice immunized with hTSHR DNA develop TSHR antibodies and, in a few mice, mild thyroiditis in association with antibodies to Tg (89). Transgenic BALB/c mice expressing low intrathyroidal levels of the human TSHR A-subunit, depleted of regulatory T cells (CD25 positive) before immunization with human TSHR-A-subunit adenovirus, developed TSHR antibodies and (unexpectedly) TgAb- and TPOAb-associated with massive thyroiditis and hypothyroidism (90). Incidentally, anti-CD25-treated BALB/c mice transplanted with TUBO tumor cells developed antitumor responses together with antithyroid immunity (98).

Of particular interest are observations of *spontaneous* antibody epitope spreading. NOD.*H2^h4^* mice develop thyroid autoimmunity, a process that is enhanced by iodide in the drinking water. The first autoantibodies to appear are directed against Tg (99–101), and subsequently, TPO antibodies appear (91). A similar pattern was observed in siblings of probands with juvenile Hashimoto thyroiditis (91). Moreover, TSHR antibodies develop spontaneously in NOD.*H2^h4^* mice transgenically expressing the human TSHR A-subunit (65).

**Figure 3:**
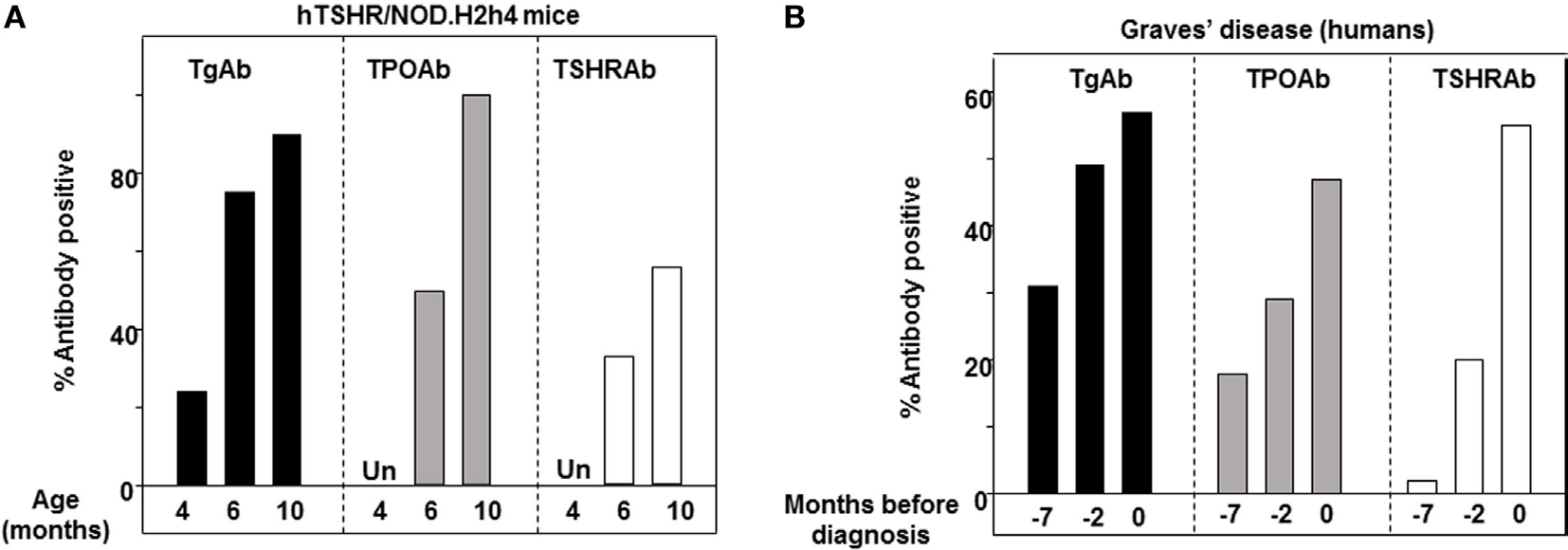
Intermolecular antigenic spreading from thyroglobulin (Tg) to thyrotropin receptor (TPO) and the thyrotropin receptor (TSHR). **(A)** Percentage of human thyrotropin receptor (hTSHR)/NOD.*H2^h4^* female mice positive for TgAb, TPOAb, and TSHRAb (TSHR binding inhibition) aged 4, 6, and 10 months. **(B)** Prediagnostic TgAb, TPOAb, and TSHRAb levels (7, 2, and 0 years) in patients with Graves’ disease [data plotted from Ref. (92)].

Combining published data from published studies (45, 102) studies, the development of autoantibodies in female hTSHR/NOD.*H2^h4^* mice permits comparing the appearance of thyroid autoantibodies over time: TgAbs are present in some 4-month-old mice, and TSHRAb and TPOAb are detectable after the age of 6 months, and all three thyroid autoantibodies were present in more mice after 10 months (Figure 3A). Turning to humans, in a study of prediagnostic markers in Graves’ patients, TPOAb and TgAb were detectable several years before TSHR antibodies (92). Importantly, although in humans the maximum percentage of positive TgAb and TPOAb in humans did not approach 100% as in NOD.*H2^h4^* mice, the time sequence of antibody reactivity to Tg, TPO, and the TSHR in transgenic NOD.*H2^h4^* mice (Figure 3A) resembles that in humans (Figure 3B).

### Intramolecular Spreading

There is less extensive evidence for intramolecular thyroid autoantibody epitope spreading (Table 2): Latrofa and colleagues demonstrated that iodine prophylaxis revealed recognition of a previously cryptic TgAb epitope, but this phenomenon may involve the iodination of Tg, thereby generating a neo-antigen (97). In addition, as already mentioned, there are rare examples of TSHR antibody switching from TBAb to TSAb associated with thyroxine therapy, or the reverse, namely TBAb to switching to TSAb [reviewed in Ref. (59)]. As would be expected for antibodies with differing functional effects, the epitopes on the TSHR ectodomain recognized by TSAb and TBAb are different (103, 104), although they interact with closely overlapping portions of the amino terminus of the TSHR A-subunit (105).

The apparent antibody epitope spreading in maternally transferred TSHR antibodies from initial TBAb to TSAb is due to progressive dilution of the high titer TBAb overpowering the lower concentrations of TSAb as human IgG is metabolized [reviewed in Ref. (59)].

## Implications of Epitope Spreading for Thyroid Autoimmunity

**Figure 4:**
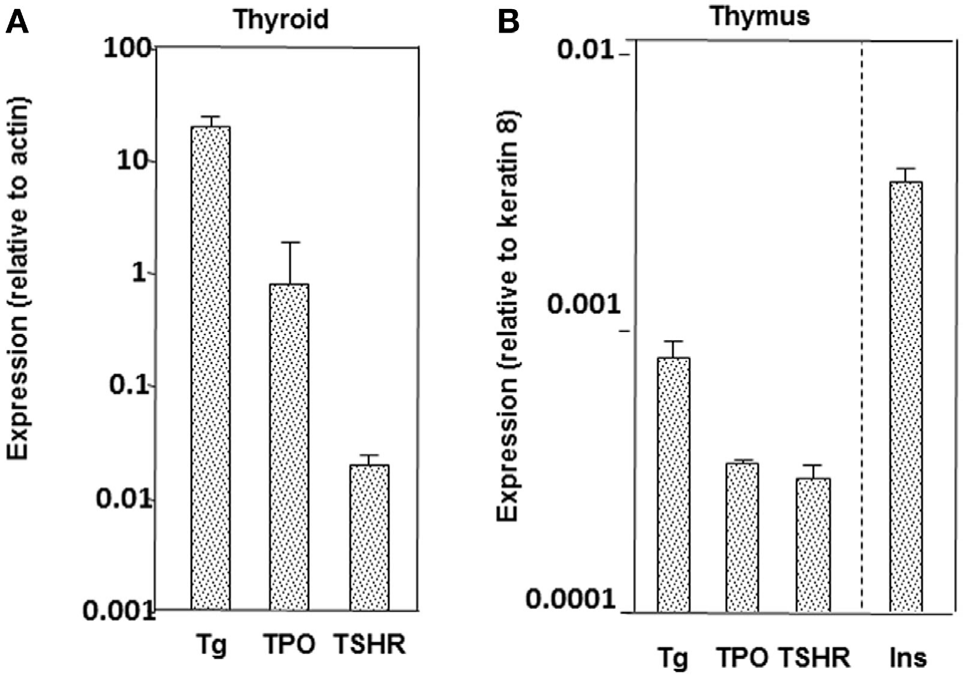
Relative expression of thyroglobulin (Tg), thyrotropin receptor (TPO), and the thyrotropin receptor (TSHR) in thyroid **(A)** and thymus **(B)** in mice (BALB/c strain). For the thymus, data are included for insulin, which is highly expressed in the thymus. Adapted with permission from the data in Ref. (107).

The phenomenon of epitope spreading provides important background information on thyroid autoantigen recognition. In particular, epitope spreading appears to be related to the amount and the size of each thyroid autoantigen, being greatest for Tg and less for both TPO and the TSHR A-subunit in the thyroid as well as in the thymus (Figure 4) [reviewed in Ref. (11)]. In part, epitope spreading involves the greater availability of peptides available to stimulate T cells and protein to stimulate B cells from the abundant, large Tg molecule compared with the more limited amount and smaller molecular size of TPO and even fewer peptides from the TSHR A-subunit [reviewed in Ref. (11)]. Central tolerance also plays a role, with responses regulated by intrathymic expression of antigens like the highly expressed transgenic human TSHR A-subunit (106).

Knowledge of the autoantigen “cascade” suggests approaches for antigen-specific treatment. For example, in NOD mice, responses against islet antigens are prevented by inducing tolerance against proinsulin but not against IGRP (8), an autoantigen recognized later in the “cascade.” In the same way, it is possible that successful induction of tolerance against Tg in NOD.*H2^h4^* mice could prevent the subsequent breakdown in tolerance to TPO (91) and perhaps even to the TSHR in hTSHR/NOD.*H2^h4^* mice (65). In a mouse model of experimentally induced thyroiditis, increasing the circulating level of Tg strengthened self-tolerance and reduced the extent of experimental thyroiditis [reviewed in Ref. (108)]. These findings suggest that increasing Tg levels could possibly regulate autoimmunity to Tg in the hTSHR/NOD.*H2^h4^* strain. However, as we previously reported, an antigen-specific approach used successfully in an induced model may not be directly applicable to a spontaneous model (45). Consequently, it may be necessary to test multiple modes of Tg presentation to downregulate the cascade of autoimmune responses to Tg, TPO, and the TSHR in hTSHR/NOD.*H2^h4^* mice.

## Conclusion

The evidence for intermolecular epitope spreading in thyroid autoimmunity is focused on autoantibodies, unlike the emphasis on T cell epitope spreading in multiple sclerosis and IDDM type 1 (or their animal models EAE and NOD mice). Unlike multiple sclerosis or EAE, original antigenic sin appears to be characteristic of thyroid autoimmunity as reflected by the recognition of an autoantibody IDR in humans and in the response of transgenic hTSHR/NOD.*H2^h4^* mice to injection with inappropriate TSHR antigen. This difference likely reflects the importance of T cell immunity in EAE/MS versus autoantibodies in thyroid autoimmunity, particularly Graves’ disease for which stimulatory autoantibodies to the TSHR are the direct cause.

Finally, the pattern observed for intermolecular epitope spreading provides the rationale for antigen-specific manipulation to block thyroid autoantibody development by inducing tolerance to the first autoantigen in the antigen cascade, namely Tg. Because of its abundance, Tg may be the autoantigen of choice to explore antigen-specific treatment to block the development of pathogenic TSHR antibodies.

## Author Contributions

SM and BR contributed to the concept, writing, and preparation of figures for this review.

## Conflict of Interest Statement

The authors declare that the research was conducted in the absence of any commercial or financial relationships that could be construed as a potential conflict of interest. The handling editor declared a past co-authorship with author SM.
